# The significant prognostic value of circulating tumor cells in triple-negative breast cancer: a meta-analysis

**DOI:** 10.18632/oncotarget.8156

**Published:** 2016-03-17

**Authors:** Yan-jun Lu, Peng Wang, Xiong Wang, Jing Peng, Yao-wu Zhu, Na Shen

**Affiliations:** ^1^ Department of Laboratory Medicine, Tongji Hospital, Tongji Medical College, Huazhong University of Science and Technology, Wuhan, China; ^2^ Institute and Department of Infectious Disease, Tongji Hospital, Tongji Medical College, Huazhong University of Science and Technology, Wuhan, China

**Keywords:** triple-negative breast cancer (TNBC), circulating tumor cells (CTCs), prognosis

## Abstract

**Background:**

The clinical validity of circulating tumor cells (CTCs) is still controversial in patients with triple-negative breast cancer (TNBC).

**Methods:**

A comprehensive literature search was performed to identify relevant articles in the PubMed, Web of Science, MEDLINE, and Embase databases through September 2015. The outcomes of interest were disease progression and overall survival. The hazard ratio (HR) and 95% confidence interval (95% CI) were considered the effect indicators and were pooled in meta-analyses under a fixed- or random-effect model according to heterogeneity.

**Results:**

Ten of the eligible studies were included for a total of 642 enrolled TNBC patients. Overall analyses revealed that the presence of CTCs predicted aggressive disease progression (HR = 2.18, 95% CI = 1.59-2.99, *P_heterogeneity_* = 0.010, *I^2^* = 52.2%) and reduced overall survival (HR = 2.02, 95% CI = 1.59-2.57, *P_heterogeneity_* = 0.169, *I^2^* = 26.6%). Further subgroup analyses demonstrated that CTC-positive patients also had poor disease progression and overall survival in different subsets, including cancer stage.

**Conclusion:**

Our meta-analysis provides strong evidence that detection of CTC in the peripheral blood is an independent prognosticator of poor survival outcomes for TNBC patients.

## INTRODUCTION

Breast cancer is the most commonly diagnosed cancer and the leading cause of cancer-related mortality in women worldwide [1]. As a distinct subtype accounting for 10-20% of all breast cancers, triple-negative breast cancer (TNBC) lacks the expression of the estrogen receptor (ER), progesterone receptor (PR), and human epidermal growth factor receptor-2 (HER-2), resulting in an aggressive phenotype for which there is no targeted therapy [2]. Traditionally, these patients are treated with a combination of surgery, chemotherapy, and radiation. Finding an effective and non-invasive method for survival prediction is clinically important to the management of TNBC patients.

Circulating tumor cells (CTCs) are cells released from the primary tumor into the peripheral blood and are considered to be a main cause of tumor metastasis [3]. In recent years, accumulating evidence has suggested the prognostic relevance of CTCs in several malignancies, such as breast cancer [4], colorectal cancer [5] and melanoma [6]. However, the prognostic value of CTC status in TNBC remains unclear. Some studies revealed that CTC status could predict poorer survival outcomes in TNBC [7–11], while other studies failed to support this conclusion [12–14]. In addition, studies by Giordano et al. [15] and Hwang et al. [16] showed that CTC status had prognostic relevance for overall survival but not for disease progression in TNBC. Karhade and his colleagues [17] found conflicting results using various cut-off criteria. Interestingly, even in the same trial, CTCs detected at different time points indicated different prognoses for survival for TNBC participants [18]. These discrepancies may result from the small sample sizes used in these studies as well as differences in the sampling times, cut-off criteria, and detection methods used.

Although a previous pooled analysis explored the prognostic relevance of CTC status in metastatic breast cancer, including TNBC [19], the effect of CTC status on non-metastatic TNBC still requires clarification. Hence, we conducted this comprehensive meta-analysis to provide a better insight into the prognostic value of CTC status for patients with TNBC. Specifically, we evaluated the potential effects of CTC status (positive vs. negative) on disease progression outcomes (disease-free survival (DFS), progression-free survival (PFS), metastasis-free survival (MFS), and time to progression (TTP)) and overall survival (OS). Furthermore, we performed subgroup analyses, including cancer stage, time points of blood collection, detection method, sample size, detection rate, and cut-off criteria, to assess the potential effect of CTC status in these different subsets.

## RESULTS

### Study characteristics

**Figure 1:**
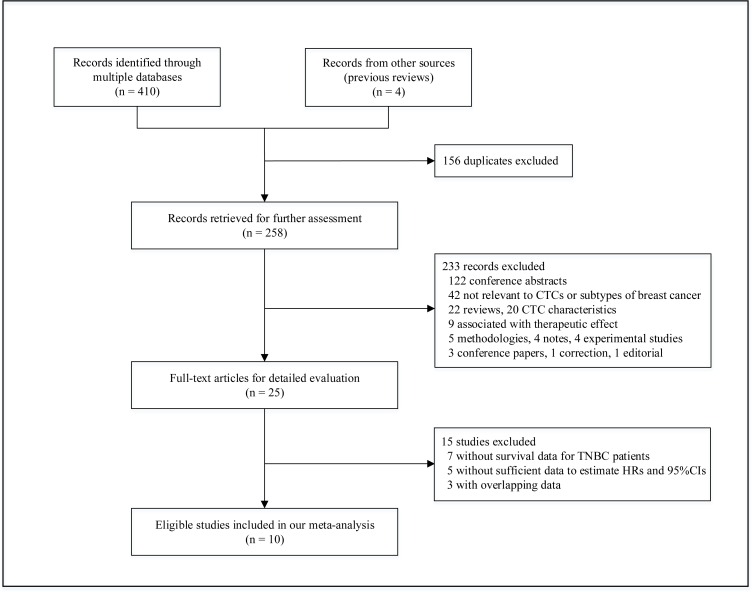
A flow chart of literature search

Figure 1 provides an overview of the process used to select the studies. A total of 414 records were initially identified by the comprehensive literature search, comprising 410 records from databases screening and 4 records from previous reviews. After reviewing the titles and abstracts, 156 duplicates were filtered out, and 233 records were subsequently excluded because they were conference abstracts, irrelevant to CTCs or subtypes of breast cancer, review articles, or experimental studies. This left 25 full-text articles for further assessment, of which 15 studies were excluded for not including survival data for TNBC patients [20–26], not providing sufficient information to extrapolate hazard ratios (HRs) and 95% confidence intervals (95% CIs) [27–31], or including patients who overlapped with those in other trials [8, 9, 32]. Finally, 10 eligible studies accounting for 642 TNBC patients were included for our meta-analysis [7, 10–18].

Early stage (M0) and metastatic stage (M1) TNBC patients were enrolled in 3 [7, 16, 17] and 7 studies [10–15, 18], respectively. Methods used for CTC detection included RT-PCR (reverse transcriptase polymerase chain reaction), CellSearch assay and IE/FC (immunomagnetic enrichment/flow cytometric) approaches. The detection rates for these methods ranged from 16% to 57%. Two main cut-off values, 5 CTC/7.5 ml and 1 CTC/7.5 ml, were used for CTC-counting methods. The characteristics of the eligible studies are summarized in Table 1.

**Table 1:** Characteristics of studies included in our meta-analysis

Study	Country	Sample size	Sampling time	Median follow-up	Stage	Detection method	Detection rate, % (n/N)	Cut-off criteria	Outcomes	HR extraction	Multivariate adjustment
Ignatiadis (2007)	Greece	77	baseline	53.5 ^a^	M0	RT-PCR	35 (27/77)	-	DFS; OS	Data extrapolated	No
Mego (2011)	USA	72	baseline	17.7 ^a^	M1	CellSearch	57 (41/72)	1 CTC/7.5ml	OS	Reported in text	No
Giordano (2012)	USA	124	baseline	24.6 ^a^	M1	CellSearch	34 (42/124)	5 CTC/7.5 ml	PFS; OS	Data extrapolated	No
Hwang (2012)	Korea	36	baseline	100.6 ^a^	M0	RT-PCR	25 (9/36)	-	MFS; OS	Data extrapolated	No
Munzone (2012)	Italy	18	baseline	42 ^a^	M1	CellSearch	22 (4/18)	5 CTC/7.5 ml	OS	Reported in text	Yes
Jiang (2013)	China	39	baseline	NR	M1	CellSearch	46 (18/39)	5 CTC/7.5 ml	PFS; OS	Reported in text	Yes
		25	mid-therapy (1st follow-up)	NR	M1	CellSearch	16 (4/25)	5 CTC/7.5 ml	PFS; OS	Reported in text	Yes
		24	mid-therapy (2nd follow-up)	NR	M1	CellSearch	17 (4/24)	5 CTC/7.5 ml	PFS; OS	Reported in text	Yes
Karhade (2014)	USA	113	baseline	40	M0	CellSearch	20 (23/113)	1 CTC/7.5 mL	PFS; OS	Reported in text	No
Peeters (2014)	Belgium	16	baseline	27.7 ^a^	M1	CellSearch	50 (8/16)	5 CTC/7.5 ml	PFS; OS	Reported in text	No
Magbanua (2015)	USA	95	baseline	26	M1	CellSearch	44 (42/95)	5 CTC/7.5 ml	TTP; OS	Reported in text	Yes
		89	mid-therapy	26	M1	CellSearch	33 (29/89)	5 CTC/7.5 ml	TTP; OS	Reported in text	Yes
		91	baseline	26	M1	IE/FC	33 (30/91)	5 CTC/7.5 ml	TTP; OS	Reported in text	Yes
		82	mid-therapy	26	M1	IE/FC	34 (28/82)	5 CTC/7.5 ml	TTP; OS	Reported in text	Yes
Paoletti (2015)	USA	52	baseline	NR	M1	CellSearch	36 (19/52)	5 CTC/7.5 ml	PFS	Reported in text	No
		52	mid-therapy (day 15)	NR	M1	CellSearch	27 (14/52)	5 CTC/7.5 ml	PFS	Reported in text	No
		49	mid-therapy (day 29)	NR	M1	CellSearch	26 (13/49)	5 CTC/7.5 ml	PFS	Reported in text	No

Abbreviations: NR, not reported; M0, non-metastasis; M1, metastasis; RT-PCR, reverse transcriptase polymerase chain reaction; IE/FC, immunomagnetic enrichment/flow cytometric; DFS, disease-free survival; PFS, progression-free survival; MFS, metastasis-free survival; TTP, time to progression.

a The median follow-up referred to all breast cancer participants.

### Overall analyses

**Figure 2:**
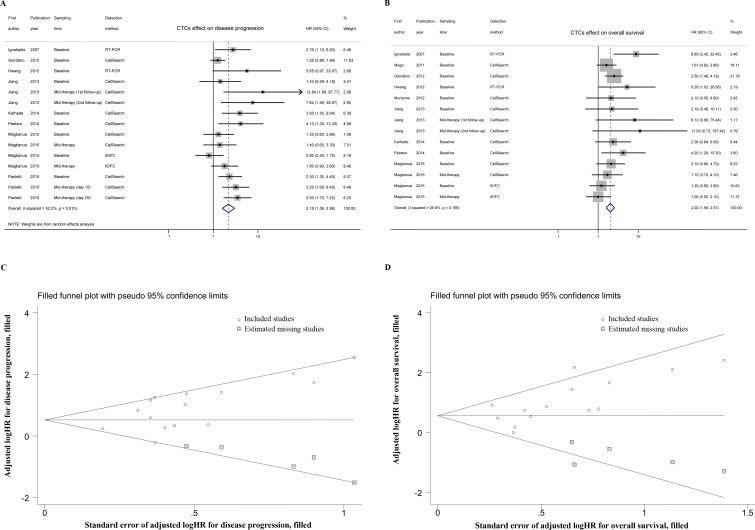
Overall analyses and imputed funnel plots Forest plots of the HRs for disease progression **A.** and overall survival **B.** in TNBC patients. Imputed funnel plots by trim-and-fill analysis for disease progression **C.** and overall survival **D.** in TNBC patients.

The HRs for disease progression (DFS, PFS, MFS, and TTP) were available in 8 studies [7, 10, 11, 14–18] accounting for 552 TNBC patients. In 3 studies [11, 14, 18], more than one HR was extracted from each trial by using multiple sampling time points or different detection methods. The overall analysis revealed that compared with CTC-negative TNBC patients, the CTC-positive patients had a higher risk of disease progression (HR = 2.18, 95% CI = 1.59-2.99, *P_heterogeneity_* = 0.010, *I^2^* = 52.2%) (Figure 2). The HRs for OS were available in 9 studies [7, 10, 12–18], accounting for 590 TNBC patients. More than one HR was extracted in 2 studies [14, 18] for the same reasons as mentioned above. The pooled results showed that CTC-positive TNBC patients also had significantly poorer outcomes than CTC-negative patients (HR = 2.02, 95% CI = 1.59-2.57, *P_heterogeneity_* = 0.169, *I^2^* = 26.6%) (Figure 2).

### Meta-regression and subgroup analyses

A significant heterogeneity among studies was observed when pooling the data for disease progression (*P_heterogeneity_* = 0.010, *I^2^* = 52.2%), so we carried out a univariate meta-regression analysis to explore the potential sources. Several covariates, including publication year, sample size, and cancer stage, were considered, and the results indicated that sample size was the main factor responsible for the heterogeneity (*P* = 0.040; Table 2).

**Table 2:** Univariate meta-regression analysis for exploring potential sources of heterogeneity

	Disease progression^a^		
	Coefficient	SE	*P*
Publication year	−0.04	0.08	0.629
Sample size	−1.17	0.51	0.040
Sampling time	0.42	0.33	0.226
Cancer stage	−0.6	0.44	0.195
Detection method	−0.31	0.38	0.432
Detection rate	−0.06	0.36	0.872
Cut-off criteria	1.05	1.08	0.355
HR extraction	−0.15	0.42	0.720
Multivariate adjustment	−0.49	0.31	0.143

Abbreviations: SE, standard error of the coefficient.

a Disease profession outcomes including disease-free survival (DFS), progression-free survival (PFS), metastasis-free survival (MFS) and time to progression (TTP). The dependent variable is the lnHR for DFS/PFS/MFS/TTP or OS of each study.

To further assess whether CTC status had prognostic value in different subsets, we also performed subgroup analyses (Table 3). We first evaluated the effect of CTC status on outcomes for different cancer stages and found that for both M0 and M1 stages, detection of CTCs predicted a poor prognosis for both disease progression (M0: HR = 3.51, 95% CI = 1.90-6.48, *P_heterogeneity_* = 0.741, *I^2^* = 0.0%; M1: HR = 1.99, 95% CI = 1.42-2.81, *P_heterogeneity_* = 0.011, *I^2^* = 55.0%) and OS (M0: HR = 4.18, 95% CI = 2.02-8.62, *P_heterogeneity_* = 0.282, *I^2^* = 21.0%; M1: HR = 1.84, 95% CI = 1.43-2.38, *P_heterogeneity_* = 0.370, *I^2^* = 7.8%). We then explored the effects of CTC status on outcomes for various sampling times. CTCs detected at baseline indicated an increased risk for both disease progression (HR = 1.86, 95% CI = 1.27-2.72, *P_heterogeneity_* = 0.041, *I^2^* = 50.4%) and poor OS (HR = 2.19, 95% CI = 1.67-2.87, *P_heterogeneity_* = 0.307, *I^2^* = 14.8%). However, CTCs detected at mid-therapy exhibited prognostic significance for disease progression (HR = 2.66, 95% CI = 1.87-3.78, *P_heterogeneity_* = 0.149, *I^2^* = 38.6%) but not for OS (HR = 1.49, 95% CI = 0.88-2.54, *P_heterogeneity_* = 0.134, *I^2^* = 46.2%). We also assessed the effects of CTC status on outcomes for various detection methods. CTCs detected by CellSearch assay or RT-PCR indicated a worse prognosis for both disease progression (CellSearch: HR = 2.37, 95% CI = 1.64-3.41, *P_heterogeneity_* = 0.018, *I^2^* = 53.4%; RT-PCR: HR = 3.22, 95% CI = 1.42-7.28, *P_heterogeneity_* = 0.480, *I^2^* = 0.0%) and OS (CellSearch: HR = 2.21, 95% CI = 1.66-2.93, *P_heterogeneity_* = 0.796, *I^2^* = 0.0%; RT-PCR: HR = 7.23, 95% CI = 2.62-19.97, *P_heterogeneity_* = 0.625, *I^2^* = 0.0%). However, we did not find any significant prognostic effect of CTCs detected using IE/FC (For disease progression: HR = 1.21, 95% CI = 0.55-2.68, *P_heterogeneity_* = 0.113, *I^2^* = 60.3%; For OS: HR = 1.09, 95% CI = 0.66-1.83, *P_heterogeneity_* = 0.728, *I^2^* = 0.0%). In addition, the effects of CTC status on outcomes were evaluated separately for different sample sizes, detection rates, and cut-off criteria. The stratified results showed that compared to CTC-negative patients, CTC-positive patients had a higher risk for both disease progression and OS in these subgroups (Table 3).

**Table 3:** Subgroup analyses of the potential effect of CTCs on survival outcomes in TNBC patients

	Disease progression^a^				Overall survival			
	n	HR (95% CI)	*P*_*heterogeneity*_	*I*^*2*^ (%)	n	HR (95% CI)	*P*_*heterogeneity*_	*I*^*2*^ (%)
Cancer stage								
M0	3	3.51 (1.90-6.48)	0.741	0.0	3	4.18 (2.02-8.62)	0.282	21.0
M1	12	1.99 (1.42-2.81)	0.011	55.0	11	1.84 (1.43-2.38)	0.370	7.8
Sampling time								
baseline	9	1.86 (1.27-2.72)	0.041	50.4	10	2.19 (1.67-2.87)	0.307	14.8
mid-therapy	6	2.66 (1.87-3.78)	0.149	38.6	4	1.49 (0.88-2.54)	0.134	46.2
Detection method								
CellSearch	11	2.37 (1.64-3.41)	0.018	53.4	10	2.21 (1.66-2.93)	0.796	0.0
IE/FC	2	1.21 (0.55-2.68)	0.113	60.3	2	1.09 (0.66-1.83)	0.728	0.0
RT-PCR	2	3.22 (1.42-7.28)	0.480	0.0	2	7.23 (2.62-19.97)	0.625	0.0
Sample size								
< 30	3	5.96 (2.53-14.04)	0.595	0.0	4	3.99 (1.73-9.18)	0.636	0.0
≥30	12	1.93 (1.43-2.61)	0.033	47.7	10	1.90 (1.47-2.44)	0.153	31.9
Detection rate (%)								
< 35	10	2.32 (1.49-3.63)	0.003	64.5	9	1.89 (1.40-2.56)	0.222	24.9
≥35	5	2.09 (1.44-3.04)	0.460	0.0	5	2.27 (1.52-3.39)	0.161	39.1
Cut-off criteria								
1 CTC/7.5 ml	12	1.99 (1.42-2.81)	0.011	55.0	10	1.91 (1.43-2.54)	0.307	14.8
5 CTC/7.5 ml	0	-	-	-	2	1.76 (1.07-2.89)	0.525	0.0

Abbreviations: IE/FC, immunomagnetic enrichment/flow cytometric; RT-PCR, reverse transcriptase polymerase chain reaction.

a Disease profession outcomes including disease-free survival (DFS), progression-free survival (PFS), metastasis-free survival (MFS) and time to progression (TTP).

### Sensitivity analyses and publication bias

For disease progression, one-way sensitivity analyses indicated the stability of our pooled results (Supplementary Table 1). Although publication bias existed (*P_Begg_* = 0.042, *P_Egger_* = 0.011), the trim-and-fill analysis revealed that after incorporating 5 additional studies, the funnel plot was symmetrical, and the adjusted pooled HR was still similar to that in the main meta-analysis (Figure 2; HR = 1.75, 95% CI = 1.27-2.41). In addition, one-way sensitivity analyses for OS also confirmed our robust results (Supplementary Table 1), and the trim-and-fill analysis indicated that despite publication bias (*P_Begg_* = 0.012, *P_Egger_* = 0.027), the adjusted pooled HR continued to show a significant association between CTC status and OS (Figure 2; HR = 1.75, 95% CI = 1.39-2.20).

## DISCUSSION

In this current meta-analysis, we performed a comprehensive literature search and provided clear evidence that CTCs detected in the blood can predict aggressive disease progression and poor OS for TNBC patients. Compared with a previous pooled analysis [19], our work not only included more subjects to support the clinical validity of CTC status in metastatic TNBC but also provided clear evidence to confirm the prognostic value of CTC status in non-metastatic TNBC. Moreover, we thoroughly evaluated the effects of CTC status on various sampling times, sample sizes, detection rates, and cut-off criteria. To the best of our knowledge, this is the first meta-analysis to comprehensively assess the prognostic value of CTC status for the TNBC phenotype.

When pooling the disease progression data, we observed significant heterogeneity among studies. The meta-regression analysis identified sample size as the primary source of this heterogeneity. However, the heterogeneity decreased or even disappeared after dividing subjects into small (*n* < 30) and large sample groups (≥30). To explore the clinical utility of CTC status in TNBC, we also carried out subgroup analyses using different stratified factors. Although M0 and M1 patients had very different prognoses, the presence of CTCs indicated worse survival outcomes for TNBC. CTCs detected at baseline or mid-therapy indicated a poor disease progression outcome. However, CTCs detected at mid-therapy failed to significantly predict poor OS, which may have been due to the insufficient statistical power. RT-PCR and CellSearch assay are two widely used methods for CTC detection. Our meta-analysis showed that CTCs detected by these two methods had a significant prognostic effect. However, for IE/FC, a new method developed by Magbanua et al. [14], the pooled HRs were not pronounced. Considering that the IE/FC group only included one study [14], more research is required to assess the effectiveness and stability of this method. The results from other subgroup analyses demonstrated that for different sample sizes, cut-off criteria, and detection rates, CTC-positive TNBC patients all had poor survival outcomes, suggesting that CTC status has a stable prognostic value.

Some limitations of this meta-analysis should be acknowledged. First, our meta-analysis was based on a comprehensive literature search, but we omitted data from several trials that did not provide sufficient information for HR extraction [27–31]. Although we were not able to extract HRs from these excluded trials, most of them suggested that CTC status had a significant prognostic value. Second, we did not acquire individual patient data from the included studies. These data, if available, might further improve the accuracy and stability of our pooled estimates. Third, obvious heterogeneity among studies was found for disease progression in our meta-analysis. Although the meta-regression analysis identified sample size as the only significant heterogeneous factor, variability in other influencing factors (e.g., study design and measurement of end points) might have contributed to the heterogeneity. Additional large-scale homogeneous studies are warranted to validate the clinical power of CTC status. Finally, the identified publication bias is of concern. We attempted to find all relevant articles, but unavoidably, some studies were likely omitted due to publication status or language restrictions. However, the trim-and-fill analysis showed that CTC status was still significantly associated with survival outcomes even when these “missing” studies were incorporated.

In summary, our meta-analysis provides strong support that detection of CTCs in the peripheral blood is an effective and promising predictor of a poor prognosis for TNBC patients. Regardless of whether CTCs are detected in an early stage or in metastatic patients, CTC status may serve as a useful tool to guide the clinical management of TNBC in the coming future. To improve the utility of CTC status in the management of TNBC, additional studies should be performed to further validate the prognostic power of CTCs detected during or after therapy and to develop a universally accepted standard method for CTC detection that has considerable sensitivity and stability.

## MATERIALS AND METHODS

### Literature search

A comprehensive electronic search was carried out using the PubMed, Web of Science, MEDLINE and Embase databases without any restriction (up to September 2015). The search items included various combinations of “breast”, “cancer”, “neoplasm”, “carcinoma”, “malign*”, “triple negative” and “circulating”. The reference lists of the retrieved articles and reviews were also checked manually for potentially relevant studies. Only articles written in English published in peer-reviewed journals were included.

### Study selection

Studies were considered eligible if they fulfilled all the following criteria: (1) retrospective or prospective cohort studies; (2) investigated the progression or survival of TNBC patients stratified by CTC status; (3) reported HRs and 95% CIs or provided sufficient information to extrapolate them. For studies with overlapping data, we only kept the study with the larger sample size. The process was performed independently by two authors, and any discrepancy was resolved by discussion or consultation with a third party if required. We documented the process *via* a flow chart as recommended by the PRISMA statement [33] (Supplementary Table 2). We did not assign a quality score to each study because no such score assessment has received a general consensus for use in non-randomized prognostic studies. Instead, we performed the widely recommended subgroup and sensitivity analyses to determine the potential effects of CTC status on the prognosis of TNBC patients.

### Data collection

Two of the authors independently collected the following data from each eligible study: first author's name, publication year, country, number of subjects analyzed, cancer stage, median follow-up, timing of blood collection, detection method, detection rate, and cut-off value for CTC status. We also recorded the prognostic outcomes (DFS, PFS, MFS, TTP, OS, survival curves, HR, and 95% CI, if available), regardless of whether they were tested by multivariate analysis. For one study [12], the reported HR indicated the CTC-negative rather than the CTC-positive arm. Thus we recalculated this HR by taking its reciprocal to maintain consistency with the other studies. When more than one blood sample per subject was collected at different time points, each sampling time point was documented and categorized as “baseline” or “mid- or post-therapy”. When more than one method was applied to detect CTCs, all results were considered as independent data sets. Any discrepancy was resolved by discussion or consultation.

### Statistical methods

The HR and 95% CI were directly recorded from each included study or extrapolated as suggested by methods of Parmar [34] and Tierney [35]. We pooled these HRs using a fixed- or random-effect model according to heterogeneity [36]. Heterogeneity among studies was tested using Cochran's Q test and quantified by the *I^2^* index, which is considered significant if *P* < 0.10 or *I^2^* > 50 % by convention [37]. We then performed a meta-regression analysis to investigate the potential causes of heterogeneity. Subgroup analyses were also conducted to determine the potentially prognostic effect of CTC status. In addition, publication bias was evaluated with Begg's and Egger's test, and its influence on the pooled HR was assessed by the “trim-and-fill” method [38–40]. To evaluate the stability of the pooled results, we carried out a one-way sensitivity analysis by recalculating the pooled HR after excluding each study in turn. All statistical tests were performed using Stata 12.1 software (College Station, TX, USA).

## SUPPLEMENTARY MATERIAL TABLES


